# “A Body Shape Index” in Middle-Age and Older Indonesian Population: Scaling Exponents and Association with Incident Hypertension

**DOI:** 10.1371/journal.pone.0085421

**Published:** 2014-01-15

**Authors:** Yin Bun Cheung

**Affiliations:** 1 Center for Quantitative Medicine, Duke-National University of Singapore Graduate Medical School, Outram Park, Singapore; 2 Department of International Health, University of Tampere, Tampere, Finland; College of Pharmacy, University of Florida, United States of America

## Abstract

**Background:**

“A Body Shape Index” (ABSI) is a recently proposed index that standardizes waist circumference for body mass index (BMI) and height. This study aims to: (a) examine if the ABSI scaling exponents for standardizing waist circumference for BMI and height are valid in middle-aged and older Indonesian population, and (b) compare the association between incident hypertension and ABSI and other anthropometric measures.

**Methods and Findings:**

The Indonesian Family Life Survey Wave 3 measured anthropometric variables and blood pressure of 8255 adults aged between 40 to 85 years in 2000. The relationship between two anthropometric quantities, e.g. weight (w) and height (h), can be expressed as the power law-equivalent 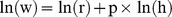, where p = 2 is the scaling exponent in the derivation of the BMI and can be estimated by linear regression analysis. This was extended to the regression analysis of the log-transformed waist circumference, weight and height to establish the scaling exponents in the ABSI. The values for men were similar to those developed by the previous American study, which were 2/3 (BMI) and 1/2 (height). Those for women were somewhat smaller, at 3/5 (BMI) and 1/5 (height). The original (American) ABSI leads to mild negative correlation with BMI (−0.14) and height (−0.12) in the female population. Analysis of the development of hypertension between Waves 3 and 4 (average interval 7.5 years) in relation to ABSI measured at Wave 3 showed stronger association if the locally derived (Indonesian) scaling exponents were used. However, both versions of the ABSI were less associated with incident hypertension than waist circumference and BMI.

**Conclusions:**

The values for the scaling exponents for ABSI are roughly similar between the American population and the middle-aged and older Indonesian population, although larger discrepancy was found in women. The ABSI is less associated with incident hypertension than waist circumference and BMI.

## Introduction

High blood pressure and overweight and obesity are major risk factors of preventable death in high and middle income countries [1]. The World Health Organization (WHO) defined overweight and obesity as body mass index (BMI) ≥25 and ≥30, respectively [2]. Useful though it is, the BMI is a crude index of weight for height. Abdominal obesity is known to be a risk factor of cardiovascular and metabolic diseases [3]–[6]. BMI does not distinguish abdominal obesity from other types of obesity. Waist circumference (WC) and waist-to-hip ratio (WHR) have been used as measures of abdominal obesity [3], [4], [6], [7]. Yusuf et al. found WHR more predictive of myocardial infarction than BMI in a case-control study of participants from 52 countries [6]. Ostchega et al. found WC associated with hypertension after adjusting for BMI in a cross-sectional study of American adults [8]. However, cross-sectional studies of Chinese population found BMI, WC and WHR similar in degree of association with the prevalence of hypertension [4], [9]. A longitudinal study of Mauritians also found BMI and indicators of abdominal obesity equally predictive of incident hypertension [10].

Recently, Krakauer and Krakauer proposed a new index called “A Body Shape Index” (ABSI) [11]. Using the same statistical principle that Benn used to develop the BMI [12], they standardized WC for weight and height, which is statistically equivalent to standardizing WC for BMI and height. In the American population sample they studied, 
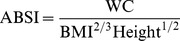
(1)


The use of the scaling exponents (or exponents in short) 2/3 and 1/2 in the denominator makes ABSI uncorrelated to BMI, weight and height. This follows the same logic that the use of the exponent 2 in the denominator of BMI makes BMI uncorrelated to height [7], [12]. This lack of correlation is an important property in the development of scientifically valid ratio indices. Without this property, an apparent association between a ratio index and a health outcome may be confounded by the variable(s) in the ratio's denominator. In the context of ABSI, the concern is whether abdominal obesity has predictive ability that cannot be explained by BMI. Therefore it is important that the ABSI is uncorrelated with BMI.

Krakauer and Krakauer found ABSI more associated with mortality hazards than BMI and WC did [11]. In a study of Portuguese adolescents, BMI showed the expected positive association with blood pressure but the ABSI showed a negative association with blood pressure [13]. It is not clear why the association was negative instead of positive. This casts doubts on the usefulness of the ABSI. The relationship between health outcomes and anthropometry in Asian populations may be different from that in other populations. In 2004, the WHO Expert Consultation reviewed evidence that Asian populations have associations between BMI, body fat, and health outcomes that are different from Caucasian populations [2]. They concluded that Asian people had elevated risk of cardiovascular and metabolic diseases even at BMI levels lower than the WHO recommended cut-off for overweight. Less is known about whether Asians are also different in terms of association between health outcomes and abdominal obesity. Within Asia, there is substantial variability in lifestyle and nutritional status. The economic development in north-eastern Asia may be leading to less traditional lifestyle and nutritional status. The population of Indonesia, the world's fourth largest population, has relatively lean body size. This further casts doubt on the applicability of the ABSI for understand important disease risks this large population.

This study aims, firstly, to examine whether the exponents derived from the American population sample are valid for Indonesia. Secondly, it compares the relative strength of association between anthropometric indices and incident hypertension.

## Methods

This is a secondary analysis of publicly available anonymized data. The analysis was approved by the Institutional Review Board of the National University of Singapore. The Indonesian Family Life Survey (IFLS) is a longitudinal, observational study of households sampled from 13 of the nation's 26 provinces, where about 83% of the population live [14]–[16]. Wave 3 (IFLS3) and Wave 4 (IFLS4) of the survey took place between June and December 2000 and between November 2007 and April 2008, respectively. The average interval between the two waves was 7.5 years. They included measurements of blood pressure, WC, hip circumference (HC) and other physical health variables of participants who were 40 years old or above and non-pregnant.

Anthropometry and blood pressure were measured by nurses of the research team. They were trained by the University of Gadjah Mada School of Public Health to conduct the measurements [14]. Weight was measured by SECA 890 scales, height by Shorr measuring boards, and waist and hip circumferences by measuring tapes. Weight was recorded to the nearest 0.1 kg; length-based height and circumferences were recorded to nearest 0.1 cm. Blood pressures were measured using Omron digital devices while the participants were in a seated position. In IFLS3 they were measured once at the beginning of the health interview. In IFLS4, blood pressure was taken at the beginning of the health interview plus two more subsequent time points during the interview. For consistency between IFLS3 and IFLS4, only the first measurement was used in this analysis. Hypertension was defined as systolic blood pressure (SBP) ≥140 or diastolic blood pressure (DBP) ≥90 or currently taking hypertension medication. Incident hypertension was defined as non-hypertensive at IFLS3 and hypertensive at IFLS4.

In allometric analysis, the relationship between two quantities, e.g. weight (w) and height (h), can be expressed according to the power law 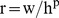, where p = 2 is the exponent in the derivation of the BMI. The relationship is equivalent to 
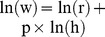
 [7], [12]. Therefore, the exponent can be estimated by linear regression analysis of the log-transformed variables. Krakauer and Krakauer applied the same principle to analyze the relationship between three quantities to develop the ABSI [11]. I used Least squares regression analysis to relate the log-transformed WC to log-transformed weight and height. All log-transformation used natural logarithm. The coefficients b1 and b2 in the regression equation
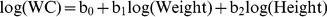
(2)are the exponents in the form of equation (1) but having weight and height instead of BMI and height in the denominator. WC and height are both in meters in this equation. These values make the index uncorrelated with weight and height. Similar to Krakauer and Krakauer, the coefficient were rounded to ratios of small integers for ease of use. Then, the index is re-expressed by replacing weight by 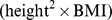.

Since the metrics of the anthropometric indices were not comparable and the values may be related to age and sex, z-score were calculated by standardizing the variables for age and sex using the exponential-normal model of Royston and Wright [7], [17], [18]. Logistic regression models with incident hypertension as dependent variable and an anthropometric z-score index as independent variable were estimated to quantify their association as odds ratio (OR). Receiver operating characteristic (ROC) analysis was used to assess the ability of each anthropometric z-score in differentiating persons who did or did not develop incident hypertension. Area under the ROC curve (AUC) was calculated and compared by a non-parametric test [19].

## Results

### Descriptive summary

There were 11810 non-pregnant adults aged 40 to 85 in the IFLS Wave 3 households, of whom 8255 participated in the physical measurements and have at least weight measured. Two hundred and forty one participants were excluded from the analysis due to missing values in blood pressure (n = 30) or anthropometry (n = 8), or outliers in anthropometric variables in univariate and bivariate (scatter-plot) examinations (n = 203; BMI<15 or >40, BMI>20 but WC<55 cm, BMI>25 but WC<60 cm, WC>130 cm, weight<30 kg, and HC<60 or >140 cm). The analysis of the exponents using IFLS3 thus involved 8014 adults.

The mean (SD) age of the participants was 52 (11) years; 51.9% were female. Summary statistics of anthropometric indicators are shown in Table 1. The population was relatively lean but diverse. While mean BMI (22.1) was normal, 18.0%, 17.7% and 3.9% of the participants were underweight, overweight or obese, respectively (Table 2). The distribution was different between genders (P<0.001). Overweight and obesity was more common in women.

**Table 1:** Descriptive summary of the participants in 2000.

Indicators	All (n = 8014)	Men (n = 3857)	Women (n = 4157)
	Mean	SD	Mean	SD	Mean	SD
Weight (kg)	52.6	10.8	55.1	10.3	50.3	10.7
Height (cm)	154.2	8.2	160.0	6.1	148.7	5.7
BMI	22.1	3.9	21.4	3.4	22.7	4.3
WC (cm)	77.3	10.7	77.5	10.1	77.2	11.2
HC (cm)	88.5	8.4	87.3	7.4	89.6	9.0
WHR	0.87	0.08	0.89	0.07	0.86	0.08
ABSI (A)	0.0795	0.0062	0.0796	0.0051	0.0795	0.0071
ABSI (I)	0.0954	0.0171	0.0796	0.0051	0.1100	0.0096

ABSI (A): ABSI as proposed by Krakauer and Krakauer (2012), see equation (1)

ABSI (I): ABSI with locally derived exponents, see equation (3)

**Table 2:** Weight status in 2000.

Weight category	BMI	All (n = 8014)	Men (n = 3857)	Women (n = 4157)
Underweight	<18.5	18.0%	18.8%	17.4%
Normal	18.5 to <25	60.3%	66.2%	54.8%
Overweight	25 to <30	17.7%	13.5%	21.7%
Obese	≥30	3.9%	1.6%	6.2%

### Scaling exponents

Table 3 shows the results of regression analysis of the log-transformed WC (m) in relation to weight (kg) and height (m). In the whole population sample, the exponents for weight and height were 0.632 and −0.801, respectively. They were quite close to the 0.681 and −0.814 reported by the American study. Krakauer and Krakauer rounded their coefficients to ratios of small integers: 2/3 and −5/6, respectively. However, looking at the analysis by gender, the relation depended on gender. Tests for interaction between gender and weight (P<0.001) and height (P = 0.009) showed statistical significance. The pattern of trivariate association and regression coefficients in men was roughly similar to those reported in the American study, but those in women were more different. As a secondary analysis, a locally derived ABSI (I) was generated as:
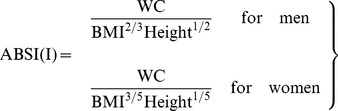
(3)


**Table 3:** Coefficients (standard errors) of regressing log waist circumference (meter) upon log weight (kg) and log height (meter).

Independent variables	All (n = 8014)	Men (n = 3857)	Women (n = 4157)
log(Weight)	0.632 (0.005)	0.679 (0.007)	0.608 (0.007)
log(height)	−0.801 (0.019)	−0.808 (0.033)	−0.947 (0.040)
Constant	−2.412 (0.017)	−2.596 (0.023)	−2.264 (0.025)

For men, equation (3) is the same as in equation (1) [11]. For women, the regression coefficients 0.608 and −0.947 were rounded to 3/5 and −1. Then weight was replaced by 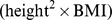 and the expression re-arranged to equation (3). For brevity, the original (American) ABSI using equation (1) and the local (Indonesian) ABSI using equation (3) are referred to as ABSI (A) and ABSI (I), respectively hereafter. The mean and SD of ABSI (A) and ABSI (I) are shown in Table 1. ABSI (I) was visibly different between men and women.

ABSI (A) was moderately correlated with WC (0.49) and strongly with WHR (0.74), although it had only weak correlation with BMI (−0.07) and almost no correlation with height (−0.03). Table 4 shows the correlation by gender. Despites its nice properties in the overall sample and in men, ABSI (A) were negatively correlated with BMI (−0.14) and height (−0.12) in women (each P<0.01). In contrast, ABSI (I) was almost free of correlation with BMI and height in women (each P>0.01).

**Table 4:** Correlation coefficients between ABSI and anthropometric indicators.

Indicators	Men (n = 3857)	Women	(n = 4157)
	ABSI (A)	ABSI (A)	ABSI (I)
ABSI (I)		0.98	
WC	0.53	0.47	0.61
WHR	0.68	0.79	0.83
HC	0.21	−0.05	0.12
BMI	0.05	−0.14	0.02 *
Weight	0.06	−0.16	0.03 *
Height	0.03 *	−0.12	0.03 *

P>0.01; all other P<0.01

ABSI (A): ABSI as proposed by Krakauer and Krakauer (2012), see equation (1)

ABSI (I): ABSI with locally derived exponents, see equation (3)

### Association with hypertension

Among 4597 participants who were not hypertensive at IFLS Wave 3, 3788 (82.4%) were measured again for blood pressure in IFLS Wave 4. The number who became hypertensive was 1448/3788 (38.2%). The anthropometric indices were standardized for age and sex to form z-scores before ROC analysis. This removed potential confounding by age and sex and put all the indices on comparable metrics. Using Royston and Wright's fractional polynomial model, it was found that log-transformation of age was needed to capture the relationship between age and ABSI (A), ABSI (I) and WC. For the other anthropometric variables, age entered Royston and Wright's exponential-normal model without transformation for generation of z-scores. Table 5 shows the logistic regression analysis results. WC and BMI had the strongest association with hypertension in terms of OR (1.32). ABSI (A) and ABSI (I) had the smallest and second smallest ORs (1.11 and 1.13, respectively). Similar findings were obtained by genders (details not shown).

**Table 5:** Odds ratios (OR) estimated from logistic regression analysis and area under curve (AUC) estimated from receiver operating characteristic analysis of incident hypertension in relation to anthropometric indices in age-and-gender standardized z-scores (n = 3788).

Predictors	OR	(95% CI)	AUC	(95% CI)
ABSI (A)	1.11	(1.04–1.18)	0.537	(0.518–0.556)
ABSI (I)	1.13	(1.05–1.20)	0.542	(0.523–0.561)
WC	1.32	(1.23–1.41)	0.579	(0.560–0.598)
WHR	1.21	(1.14–1.30)	0.556	(0.540–0.577)
HC	1.27	(1.19–1.36)	0.570	(0.551–0.589)
BMI	1.32	(1.24–1.41)	0.581	(0.562–0.580)
Weight	1.28	(1.19–1.37)	0.570	(0.551–0.589)

ABSI (A): ABSI as proposed by Krakauer and Krakauer (2012), see equation (1)

ABSI (I): ABSI with locally derived exponents, see equation (3)

In ROC analysis, ABSI (A) and ABSI (I), respectively, gave the smallest and second smallest AUC (0.537 and 0.542) in differentiating persons who did or did not subsequently develop hypertension (Table 5). The global null hypothesis of all indices having the same AUC was rejected (P<0.001). In pairwise comparison, ABSI (I) and ABSI (A) had significantly smaller AUC than each of the other indices (each P<0.05). BMI and WC, respectively, gave the largest and second largest AUC (0.581 and 0.579). There was no statistically significant difference between BMI and WC (P = 0.765). Similar findings were obtained in men and women (details not shown).

## Discussion

Weight- and length-based anthropometry measurement is an important tool in public health and clinical management of growth problems and obesity. Despite their lack of technological sophistication, they are portable, inexpensive and known to predict cardiovascular and metabolic disease outcomes. They are particularly useful in population-based studies that involve large-scale operation. BMI is a crude measure of weight for height. There have been calls for alternative measures of overweight and obesity. Indices that relate to abdominal obesity have received particular attention. Waist circumference and waist-to-hip ratio have been shown to predict health outcomes [3]–[4], [6], [8]. The ABSI is a recently proposed measure that standardizes WC to weight and height. This adds a potentially valuable tool for the assessment of population at risk and has led to substantial international interest. Nevertheless, whether the same coefficients could be used to properly standardize WC for weight and height in populations that may not have the same pattern of body size and shape is unknown.

In transitional economies, the population may be relatively lean. But at the same time they are diverse in terms of the co-existence of under- and over-weight in the same communities or households [20]–[21]. The Indonesian population is relatively lean even among Asian standard, e.g. lower mean BMI and WC than the Chinese population in south [22] and north China [4]. Nevertheless, there was a substantial proportion of people who were overweight or obese. Drawing on data from a large-scale, nationally representative population survey, it is shown that the values for the exponents for calculating the ABSI in Indonesia were somewhat smaller than those derived from the American study. In particular, in the study of women, the original (American) ABSI induced negative association with BMI, weight and height. This suggests that as BMI and height increase, WC increases more in the participants of the American than the Indonesian study. Abdominal obesity has not yet become as important in this population as in the American population. This may have partially contributed to the limited usefulness of the ABSI in Indonesia. A comprehensive understanding of the weak association between ABSI and hypertension is not yet available. The use of the original (American) ABSI in Indonesia over-standardizes for BMI, weight and height, leading to negative correlation with them. A study of Portuguese adolescents showed a negative correlation between ABSI and blood pressure [13]. There is not sufficient information available for us to understand why. Stages in pubertal development may have affected the association. But it is also possible that the exponents were not appropriate in that population.

A limitation of the study was that only one reading of blood pressure was used to define hypertension. This may have over-estimated the prevalence of hypertension. However, this study does not concern hypertension per se. It concerns the anthropometric indices and their relative performance in terms of association with hypertension. Since the analyses of the indices were equally influenced by the BP measurement, the comparison was fair. The findings should not be interpreted beyond this.

While the use of the same index has the advantage for international comparison, it incurs a cost of not being optimal in a local population. Researchers should balance these two concerns in their analysis. It is possible to use two versions of the same general index structure for different groups, e.g. the ABSI (I) uses the same structure but different coefficients for men and women. This is not a new idea. For example, the Ponderal Index uses an exponent 3 to standardize weight for supine length in neonates. But neonates born at different gestational ages may need different exponents. So the exponent may be derived specifically according to gestational age under the same general index structure [23].

The relationship between cardiovascular and metabolic risk factors and anthropometric indices has been explored over the years, with BMI being a key measure. Recently, WHR and ABSI were found to be more predictive of myocardial infarction and premature death, respectively, than BMI [6], [11]. They highlighted not only the crudeness of the BMI but also the importance of abdominal obesity. A recent study showed that ABSI, WC and BMI had similar ability to predict diabetes onset [24]. There is also conflicting data on whether anthropometric indicators of abdominal obesity are useful in revealing hypertension risk, given the information on BMI [4], [8]–[10], [24]. In the present study, both WC and BMI were more strongly associated with the development of hypertension. In contrast, the ABSI and the version using locally derived exponents showed weaker association. In the Indonesian population at this stage of the epidemiological transition, the BMI will likely remain a useful public health and clinical indicator.
